# Validation of Continuous Monitoring System for Epileptic Users in Outpatient Settings

**DOI:** 10.3390/s22082900

**Published:** 2022-04-09

**Authors:** David Zambrana-Vinaroz, Jose Maria Vicente-Samper, Jose Maria Sabater-Navarro

**Affiliations:** Neuroengineering Biomedical Research Group, Miguel Hernández University of Elche, 03202 Elche, Spain; jose.vicentes@umh.es

**Keywords:** ambulatory, artifacts, ear EEG, ECG, epilepsy, HRV, monitoring system, PPG, PTT, wearable

## Abstract

Epilepsy is a chronic disease with a significant social impact, given that the patients and their families often live conditioned by the possibility of an epileptic seizure and its possible consequences, such as accidents, injuries, or even sudden unexplained death. In this context, ambulatory monitoring allows the collection of biomedical data about the patients’ health, thus gaining more knowledge about the physiological state and daily activities of each patient in a more personalized manner. For this reason, this article proposes a novel monitoring system composed of different sensors capable of synchronously recording electrocardiogram (ECG), photoplethysmogram (PPG), and ear electroencephalogram (EEG) signals and storing them for further processing and analysis in a microSD card. This system can be used in a static and/or ambulatory way, providing information about the health state through features extracted from the ear EEG signal and the calculation of the heart rate variability (HRV) and pulse travel time (PTT). The different applied processing techniques to improve the quality of these signals are described in this work. A novel algorithm used to compute HRV and PTT robustly and accurately in ambulatory settings is also described. The developed device has also been validated and compared with other commercial systems obtaining similar results. In this way, based on the quality of the obtained signals and the low variability of the computed parameters, even in ambulatory conditions, the developed device can potentially serve as a support tool for clinical decision-taking stages.

## 1. Introduction

Epilepsy is a chronic disease with an enormous social and health impact. Its worldwide incidence is around 50 cases per 100,000 inhabitants every year [1]. Although a large number of antiepileptic medicines are now available, as well as other more selective treatments such as surgery or brain stimulation, a considerable percentage of patients are not fully under control yet, and they continue to have epileptic seizures.

The lack of predictability of seizures and their possible serious consequences is, therefore, a major limitation for the independence of people with epilepsy, and it greatly impacts their quality of life. For this reason, various technologies have been developed in recent years to detect epileptic seizures in the patient’s usual environment. These technologies have the ability to provide a reliable clinical diagnosis through the collection of biomedical data about the patients’ health over a certain period of time by means of portable ambulatory systems [2]. In this way, the hospitalization costs for patients can be reduced, and the health conditions can be monitored in a home environment, away from hospitals. In addition, they allow obtaining greater knowledge about the physiological state and the daily activities of each patient in a more personalized way [3]. 

The main characteristics of portable ambulatory systems are their small size and comfort for the patient, in addition to their main objective, which is to provide information about the user’s health status through registered biomedical signals. Even though a multitude of portable devices have been developed for ambulatory monitoring, there are certain limitations and challenges regarding the recording of biomedical variables in the patient’s usual environment. Due to the fact that this environment is not a controlled scenario, such as a hospital center, and the patient carries out their daily activities while moving, some noise is generated in the signals, which significantly worsens their quality. In this sense, there can be two types of artifacts: physiological and non-physiological ones. The latter is caused by problems in the measurement equipment, such as power line interferences, whilst the former is caused by muscle activity, skin interference, or body movements [4,5]. 

Since the signals can be distorted by many artifacts which are not related to physiological conditions, this can sometimes lead to false diagnoses [6]. That is why noise filtering is a necessary step for the reliability of the data provided by these ambulatory systems. Although sometimes the noise spectrum is superimposed on the studied signal, considerably hindering its elimination, there are different signal processing techniques that can be helpful, such as digital filters, adaptive filters, wavelet transform, PCA and ICA [7,8,9,10].

Some of the most commonly involved biomedical signals when monitoring an epileptic patient are:

- Electrocardiogram (ECG): the electrical activity of the heart is measured since every time the heart beats, an electrical signal flows through it. In order to record this signal, electrodes are placed on the chest. Thanks to the ECG, several heart diseases such as arrhythmia or heart failure can be diagnosed [11].
- Photoplethysmography (PPG): this is a non-invasive optical technique that is used to detect changes in blood volume in the microvascular layer of the tissue [12]. Commonly, there are two configurations to obtain the photoplethysmography signal, one of them consisting of placing an LED diode on one side of the tissue and the photodetector on the other side of the tissue so that the photodetector measures the transmitted light. The other configuration consists of placing the LED diode and the photodetector on the same side of the tissue in such a way that the photodetector measures the reflection of light. PPG voltage signal is inversely proportional to the amount of blood flowing in the former case, and proportional in the latter case. Thus, this technique allows detection of the pulse wave that is transmitted through the blood vessels [13].
- Electroencephalogram (EEG): the electrical activity of the brain is measured. This signal is used to develop applications related to brain–machine interfaces. Some important applications are emotion recognition [14,15] neuromotor disorders [16,17] and brainwave-based control [18,19,20]. There are studies that have compared and validated the results obtained from neuropsychological tests of attention with attention level tests based on BCI systems [21]. The changes that occurred in the intensity of brainwaves of test subjects recorded while browsing different media content were analyzed in [22]. Apart from BCI systems, there are other methods of human-computer interaction, such as eye movement tracking [23]. These systems can be used in the analysis of programming technologies such as LINQ [24], thus allowing, the loading of cognition or source code and algorithm description tools for readability [25]. Moreover, EEG is an essential clinical tool to study and diagnose many neurological diseases, such as epilepsy [26,27]. In a conventional way, EEG signals are recorded by placing electrodes on the scalp. However, this is not practical for the acquisition of EEG in natural situations of daily life, in which the acquisition system must be portable, discreet and with minimal disturbance for the user. As a consequence, several ear-focused EEG solutions have been presented in recent years [28,29,30,31].

From these signals it is possible to extract information about the patient’s health status by computing certain parameters. On the one hand, heart rate variability (HRV) is the physiological phenomenon of the variation in the time interval between each heartbeat, that is, the time between an R peak and the next one of the ECG signal. Similar to many other systems (renal system, digestive system, etc.), the cardiovascular system is linked to the central nervous system [32]. Heart rate is under the control of the autonomic nervous system, which has a sympathetic and parasympathetic branch. In a condition of stress or illness, a predominance of the sympathetic nervous system arises that causes the electrical system of the heart to become unstable. Therefore, it is a marker of the relative activation state of the sympathetic-co-parasympathetic axis [33,34]. In addition, HRV parameters contain valuable information and can be used as a predictor of seizures [35,36].

Another important parameter is the pulse transit time (PTT), i.e., the time it takes for the pulse pressure waveform (PP) to propagate along the arterial tree. The PTT parameter is used as an indicator of blood pressure change [37,38]. This parameter is inversely proportional to blood pressure, because an increase in blood pressure causes the pulse wave to travel faster towards the measurement periphery, thus reducing the pulse transit time. However, when the blood pressure falls, the pulse transit time increases. It is noteworthy that seizure activity can cause both a decrease and an increase in blood pressure, probably due to stimulation or inhibition of the distinct central autonomic function by epileptic activity propagating to different neural networks of the central autonomic nervous system [39,40].

Regarding the parameters that can be used to obtain information from the EEG signal, numerous feature extraction techniques have been proposed, such as statistical features (mean, standard deviation, variance, skewness, kurtosis), spectral power, entropy, Fast Fourier Transform (FFT), Autoregressive Models (AR), Common Spatial Patterns (CSP), Spectral Graph Wavelet Transform (SGWT), Discrete Wavelet Packet Transform (DWPT), Hilbert Transform or Neural and Fuzzy Networks, as well as combinations of them [41,42,43]. There are multiple commercial ambulatory devices to obtain these signals. Smart wristbands or wristwatches, such as the AppleWatch® [44], can be used to measure the ECG signal, which is very comfortable for the user. However, these devices do not perform continuous signal monitoring. Another solution is the use of biopatches that are attached to the user’s chest for ECG measurement, e.g., the Zio Patch [45]. This type of device performs continuous signal measurement, and it can even acquire other parameters. An example is the Max-ECG-Monitor [46], which, in addition to the ECG signal, measures the user’s body temperature and motor activity. Another example of an ambulatory ECG signal measurement system is the chest bands, such as the Zephyr BioHarness [47]. These devices perform stable ECG measurement in ambulatory activities even during tasks where there is high physical intensity, and, similar to the previous ones, they can integrate the measurement of other biosignals of the user. 

In order to obtain the PPG signal, there are solutions where the device also performs the measurement on the wrist, for instance, the AppleWatch® or the Fitbit Charge® [48], which also integrates the measurement of the electrodermal activity. There are also other devices that measure the PPG signal on the ear, for example, the Cosinuss° One [49], which uses the PPG signal to calculate the HR, or on the fingers, either by means of a clamp attached to a finger such as a pulse oximeter, for example, the Nellcor Portable from Medtronic [50], or by using a ring, such as the OURA device [51]. 

Finally, the available devices for the EEG measurement employ two main solutions to position the electrodes on the user’s head: a helmet, such as the Emotiv EPOC FLEX [52], which supports up to 32 electrodes, or a headband, such as the NeuroSky MindWave [53]. The headband is less intrusive and is, therefore, more comfortable for the user. However, it is more sensitive to the user’s movement. In addition, EEG measurement in the ear, either internally (by contacting the ear canal) or externally, has also been used recently. This solution requires fewer electrodes, and it thereby involves a considerably smaller measurement area than the traditional method. An example of this approach is the cEEGrid [54], which consists of a 10-electrode array manufactured using flexible printing technology that is placed around the ear. There is also another device in the format of an earpiece (MJN-Seras®) that can record and monitor brain activity through the ear canal. Thanks to the artificial intelligence algorithms embedded in this system, it can calculate the risk of a seizure at any time, indicating it to the patient by means of a color code [55].

There are also many research projects where devices for the measurement of these signals have been developed. For example, in work developed by Masihi et al. [56], a device for continuous ECG signal measurement was presented, where two dry electrodes were integrated into the fabric of a T-shirt, and the information was sent by wireless communication to a smartphone. Another example is the work developed by Fiege et al. [57], where the authors implemented an automatic seizure detection using the pulse transit time. In [58] Juez developed a wearable system with In-Ear EEG electrodes for the monitoring of brain activities for epilepsy. The work developed by Yamakawa et al. [59] presented a wearable epileptic seizure prediction system with machine-learning-based anomaly detection of heart rate variability.

Since there are no systems capable of measuring ECG, PPG and ear EEG signals simultaneously in a static and ambulatory manner, a novel monitoring system is proposed, consisting of different sensors capable of recording these signals and storing them for further processing and analysis in a microSD card. In the Section 2, the characteristics of the proposed device are described, as well as its design and implementation. Later, the processing techniques that have been carried out to improve the signals and the algorithms used to calculate the HRV and PTT parameters robustly in both a static and ambulatory environment will be shown. Finally, the results obtained will be shown, and the quality of the recorded signals and the variability of the statically computed parameters will be discussed and compared with the results obtained in ambulatory settings. 

## 2. Materials and Methods

### 2.1. Device Description and Implementation

The ambulatory monitoring system has been designed by combining an OpenBCI board (for EEG signal acquisition) and the MAX86150 module (for ECG and PPG signals acquisition). The materials and implementation are described below.

#### 2.1.1. OpenBCI

**Figure 1:**
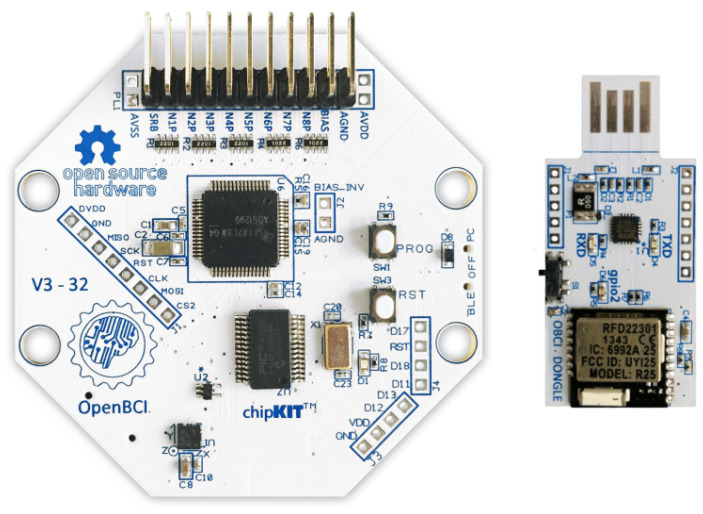
OpenBCI Cyton board and dongle.

The OpenBCI Cyton board (Figure 1) is an 8-channel neural interface used to measure and record electrical activity produced by the brain (EEG) [60]. A PIC32MX250F128B microcontroller is implemented on the board, providing plenty of local memory and fast processing speeds. In addition, the board contains:

- An integrated circuit developed by Texas Instruments for biopotential measurements with 24-bit resolution (ADS1299).
- A 3-axis accelerometer (LIS3DH).
- A module for storing data in a micro SD card.
- A radio module (to connect and communicate with a dongle connected to a computer or tablet). The application to communicate with OpenBCI is an open-source application written with Processing language.

It should be noted that the microcontroller (PIC32) operates at a voltage of 3.3 V, and it communicates with the rest of the on-board components (ADS1299, LIS3DH, microSD card) using the SPI communication protocol. 

The use of the OpenBCI device for the acquisition of the EEG is highly sustained in the literature. For example, in [61], the design and evaluation of a high-fidelity prototype that wirelessly acquires the EEG signal by implementing an OpenBCI system are presented. In addition, Refs. [62,63] also provide an analysis of the quality of the acquired signals using the ADS1299 integrated circuit capable of recording EEG signals in uncontrolled environments.

**Figure 2:**
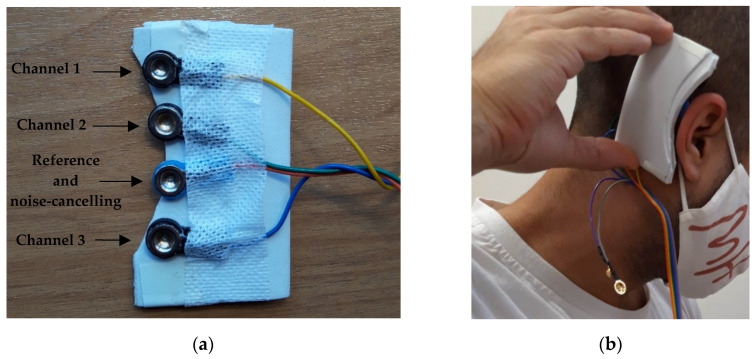
(**a**) Flexible gold cup electrode array; (**b**) Placement of the flexible gold cup electrode array behind the ear. The electrode array is held in the right place by two elastic fabric strips.

In order to be able to acquire the EEG signal from the ear and subsequently record it with the OpenBCI system, an electrode array was designed and manufactured. Its shape was adapted for placement behind the right ear (temporal lobe). The array holder was made of EVA (ethyl vinyl acetate) rubber and incorporated the holders into which four gold cup electrodes (three channels + one reference and noise-canceling) were inserted. Two elastic fabric strips were used to hold the EVA holder in the right place so that the electrodes made sufficient contact with the skin to properly acquire the EEG signal from the ear. In this way, the holder was held in place by pressing the electrodes against the patients’ skin. The gold cup electrodes used are reusable and designed to provide reliable signals. Figure 2a shows the electrodes that make up the array, and Figure 2b shows the placement of the flexible gold cup electrode array behind the ear.

#### 2.1.2. MAX86150

Maxim Integrated’s MAX86150 biosensor module [64] offers PPG and ECG measurements in a single integrated package with 16-bit resolution. The MAX86150 module combines internal LEDs, photodetectors, and an ECG Analogue Front-End (AFE) to provide highly accurate, low-power PPG and ECG signals acquisition. It was chosen because of its small dimensions (3.3 mm × 5.6 mm × 1.3 mm) and because it is capable of performing ECG signal acquisition with only two electrodes, thanks to the fact that the instrumentation amplifier in the MAX86150 has a high common-mode rejection ratio (CMRR). The MAX86150 operates on a supply voltage of 1.8 V with a separate power supply for the internal LEDs (3.3 V). Communication to and from the module is entirely via a standard I2C-compatible interface.

At the input of each of the electrodes (positive and negative), a low-pass passive *R*-*C* (*H*(ω)) filter was implemented in order to eliminate the high-frequency components associated with noise. For this purpose, a resistor (*R*) with a value of 50 kΩ and a capacitor (*C*) with a value of 10 nF was used, resulting in a cut-off frequency (*f_c_*) of 318.31, Hz as shown in the following Equations (1) and (2). This frequency is higher than the standard frequency spectrum of the ECG signal, which ranges from 0 Hz to 150 Hz [65].

(1) $$H(\omega )=\frac{1}{1+j\omega RC}=\frac{1}{{1+j\omega \times 50\times 10}^{3}{\times 10\times 10}^{-9}}$$

(2) $$fc=\frac{1}{2\pi RC}=\frac{1}{{2\times \pi \times 50\times 10}^{3}{\times 10\times 10}^{-9}}=318.31\;\mathrm{Hz}$$

**Figure 3:**
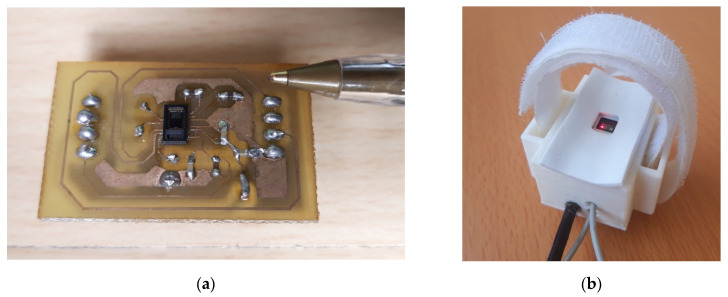
(**a**) MAX86150 sensor printed circuit board; (**b**) White housing for the MAX86150 module printed circuit board.

Figure 3a shows the circuit board of the MAX86150 module, highlighting its tiny size. A white housing for the printed circuit board was also designed (Figure 3b), which was placed on the wrist, and it was fixed with Velcro to measure PPG. In order to improve the comfort when recording the PPG signal on the wrist, the upper part of the housing was lined with EVA rubber. 

In addition, a black cable (power and communication) and two electrodes were attached to the chest for the purpose of ECG measurement. The chosen electrodes are disposable electrocardiogram electrodes in a circular shape (50 mm diameter) made of foam with a hook-and-loop connection. It is worthy to note their great durability and adherence thanks to their semi-liquid gel composition.

#### 2.1.3. Ambulatory Monitoring System

**Figure 4:**
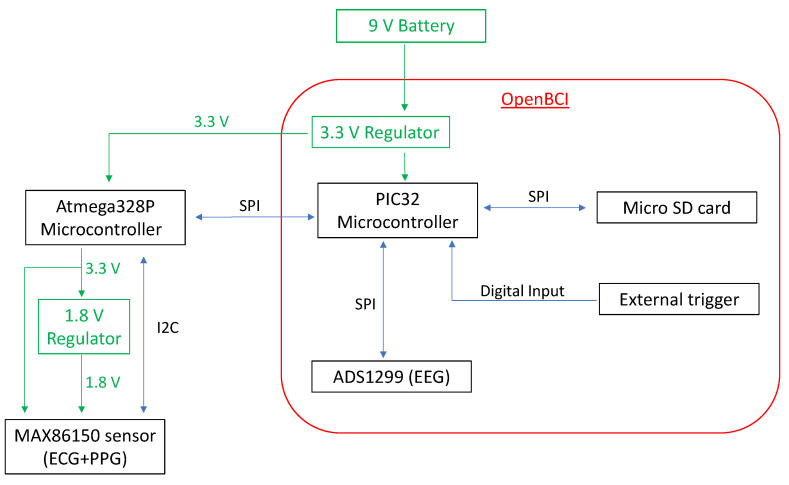
Schematic diagram of the operation and communication of the designed monitoring system.

As mentioned above, the ambulatory monitoring system was designed by combining an OpenBCI board (EEG) and the MAX86150 module (ECG and PPG). Since the PIC32 microcontroller on the OpenBCI board communicates using the SPI protocol and the MAX86150 integrated board can only communicate using the I2C protocol, direct communication is not possible. Thus, we decided to implement the communication between them by means of an Atmega328p microcontroller. In this system, Atmega328p microcontroller acts as master and communicates via the I2C protocol with the MAX86150 module, while it parallelly also acts as a slave (sending the information read by the MAX86150 module to the PIC32), and it communicates via the SPI protocol with the PIC32 microcontroller on the OpenBCI board, the latter being the master. In this way, the synchronization between the two modules is provided, which means that the information read by the sensors can be stored in the micro SD card on the OpenBCI board for subsequent analysis. The sampling frequency of the biomedical signals being monitored (ECG, PPG, and ear EEG) was set to 250 Hz. A button (external trigger) was added, which was connected to one of the digital inputs of the PIC32. When it is pressed, the system records and stores in the microSD card the external events, such as seizures, changes of activity that can trigger a seizure, etc. In this way, it is easier to differentiate and separate patient time periods and to relate the stored biomedical variables to the activities performed by the patient. Figure 4 shows the schematic diagram of the operation and communication of the designed monitoring system.

The system was powered by a 9 V battery connected to the OpenBCI board by means of a switch to turn the system on and off. The OpenBCI board has a built-in 3.3 V regulator that supplies power to the PIC32 microcontroller and its respective integrated units and modules. This regulator also supplies power to the Atmega328P microcontroller, which in turn supplies power to the MAX86150 integrated unit and to another 1.8 V regulator, which is also used to supply power to the MAX86150 integrated unit (since it needs two levels of power to be able to obtain ECG and PPG simultaneously). In order to supply 1.8 V to the MAX86150 sensor, a small printed circuit board was designed, which had a 1.8 V voltage regulator and pull-up resistors to carry out the I2C communication between the MAX86150 and the Atmega328p microcontroller. 

The PIC32 microcontroller stores the data in hexadecimal format in the microSD card in blocks, i.e., as the microcontroller receives the data from the sensors, the current data block is being built, and when the block is complete (512 bytes), the data are written to the file in the microSD card. In this way, the data are written efficiently, thus avoiding interruptions, delays, and loss of the acquired data. 

**Figure 5:**
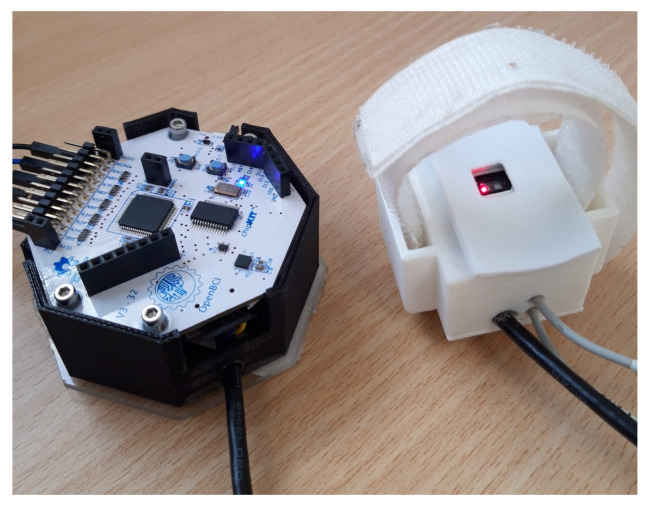
Black case in which the OpenBCI board is attached with screws and just below the board there are the power supply for the system (9 V battery) and the Atmega328p microcontroller. On the lower part of the case there are two Velcro strips so that it can be placed on a strap around the user’s waist.

A black case was designed in order to keep the whole system compact and portable. The OpenBCI board was attached to the housing with screws. Immediately below the board, there were the power supply for the system (9 V battery) and the Atmega328p microcontroller. On the lower part of the case, there were two Velcro strips so that they could be placed on a strap around the user’s waist. As can be seen in Figure 5, a black cable comes out of the Atmega328p microcontroller (inside the black case) and communicates with the MAX86150 module (white case).

To manage the recording time of the monitoring system and to be able to observe the recorded signals, a digital tablet was used, which communicated with the monitoring system only by means of a Bluetooth dongle connected to the USB port. A graphical interface was used on the tablet to interact with the designed monitoring system (the options were: start, stop and resume recording and view recorded signals). However, to carry out ambulatory recordings, it was only necessary to select the time to be monitored (which can vary from a minimum of 5 min to a maximum of 24 h) and start the recording. The user could then move away from the tablet or even turn it off, and after the selected recording time has elapsed, the data would be stored in the microSD card. 

**Figure 6:**
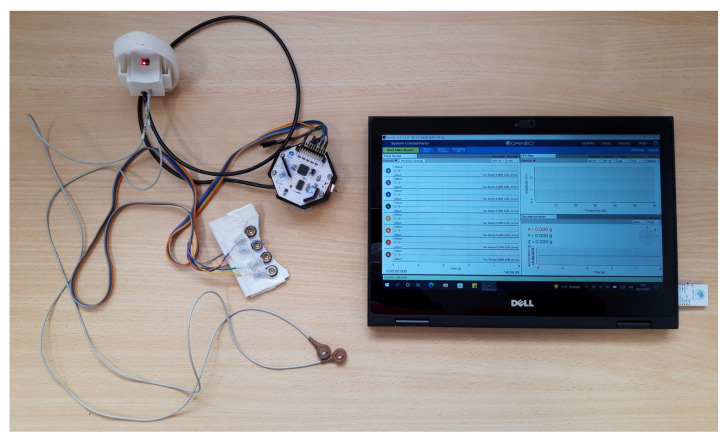
Ambulatory monitoring system with the tablet.

The Graphical User Interface (GUI) provided by OpenBCI was used to develop a customized GUI. It was designed in Processing language, which is an open-source programming language and integrated development environment based on Java. As it is open-source, it could be modified and adapted according to the needs of the ambulatory monitoring system. Figure 6 shows the complete monitoring system, including the tablet.

**Figure 7:**
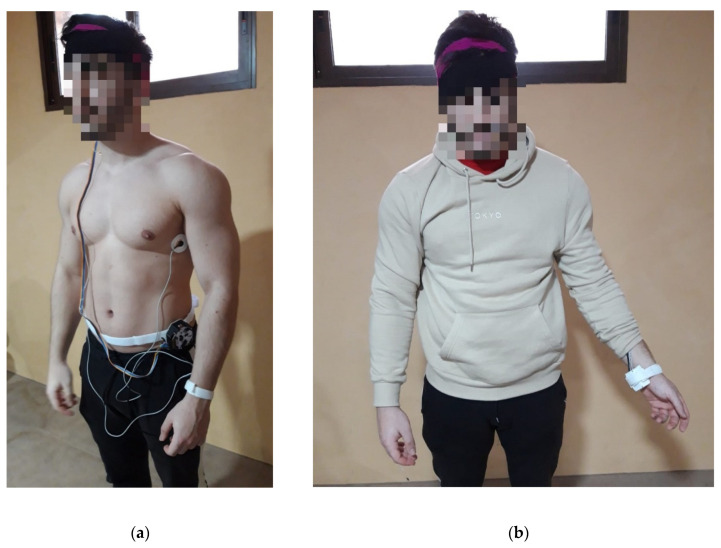
(**a**) Placement of each of the elements that make up the ambulatory monitoring system on the user; (**b**) Final arrangement of the ambulatory monitoring system on the user with clothing. It can be seen that the design is unobtrusive and produces practically no disturbance to the user’s daily activities.

As for the final arrangement of the monitoring system on the user, as mentioned above, the PPG sensor was attached to the wrist by a Velcro strap, the two ECG electrodes were placed on both sides of the chest, and the ear EEG array was attached behind the right ear by means of two elastic fabric strips. The electronics housing is attached by Velcro to a strap at user’s waist level. Figure 7 shows the final arrangement of the monitoring system on a user.

### 2.2. Static System Validation 

In order to validate the data acquired by the monitoring system, tests were made with 5 healthy volunteers (Table 1). For assessment purposes, the data were recorded statically, with the volunteers sitting when there was no movement of the user’s body; therefore, the noise-signal ratio is the lowest possible. The experimental procedure lasted for 20 min, and it was designed based on the advice of a neurology specialist. The involved activities were:

1. Eyes closed (*15 min*).
2. Opening and closing the eyes (*1 min*).
3. Hyperventilation, 15–20 breaths/min (*3 min*).
4. Light stimulation with open eyes, blinking at 2.5 Hz (*1 min*).

In order to validate and compare with other systems the signals recorded by the proposed device, two commercial devices were used in parallel during the experimentation. Firstly, Zephyr BioHarness [66,67] was used to acquire the ECG signal and to obtain the HRV values provided by the device at the end of the experiment. Secondly, the e-Health platform [68] was used to acquire the ECG and PPG signal.

#### 2.2.1. Signal Processing

**Figure 8:**
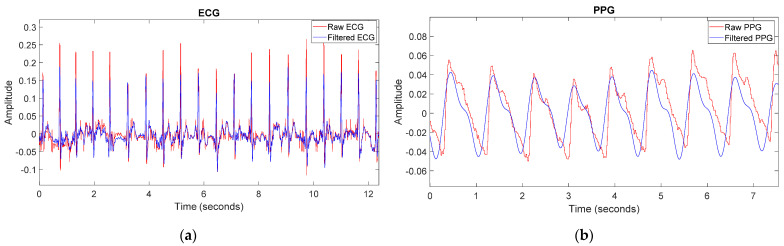
(**a**) Raw ECG signal in red and the filtered signal in blue; (**b**) Raw PPG signal in red and the filtered signal in blue.

Once the recordings had been fulfilled, the data stored in the microSD card in hexadecimal format in a text file were converted into signed decimal values and analyzed using Matlab software. In order to improve the quality of the signals and eliminate the possible noise, the signals were filtered. For the ECG signal, a bandpass filter from 0.4 Hz to 20 Hz was implemented with subsequent smoothing. Figure 8a shows the raw ECG signal in red and the filtered signal in blue. As shown, the obtained signal is devoid of noise and has a better quality. Regarding the PPG signal, a bandpass filter from 0.4 Hz to 3 Hz was used. Figure 8b shows the raw PPG signal in red and the filtered signal in blue. As it can be seen, the signal obtained is smoother, and the undesired small peaks in the raw signal have been eliminated.

From ear EEG signals, changes in brain activity can be obtained accurately, detecting different levels of stress, sleep or concentration. These signals can be broken down into four frequency components associated with mental levels [69,70]:

- Delta waves (<4 Hz): these ones have the largest wave amplitude and are related to deep sleep.
- Theta waves (4–8 Hz): these ones are related to internal cognitive tasks, imaginative abilities, reflection and sleep.
- Alpha waves (8–13 Hz): these ones predominate when the Central Nervous System is at rest, relaxed but awake and attentive.
- Beta waves (13–30 Hz): these ones are associated with external cognitive tasks, and activities related to concentration, such as solving a mathematical problem.

**Figure 9:**
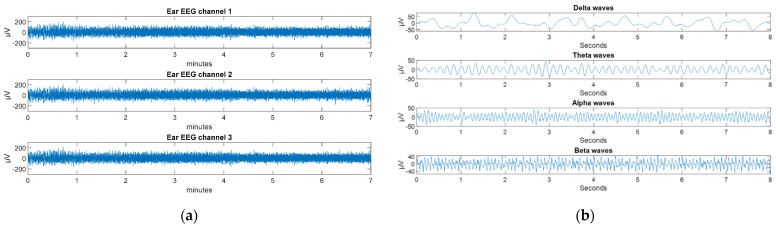
(**a**) Each of the filtered channels of the ear EEG signal; (**b**) Different waves that make up a channel of the EEG signal from the ear.

For each one of the 3 ear EEG signals obtained, first, a notch filter was used at 50 Hz (to remove any electrical interference), and then the signal was bandpass filtered from 0.5 Hz to 80 Hz. The ear EEG signals were then further bandpass filtered in a second stage to obtain specific information about the different waves that make them up: Delta, Theta, Alpha, and Beta waves. Figure 9a shows the three channels of the ear EEG signal, already filtered in first stage, whereas Figure 9b shows the different waves that make up a channel of the EEG signal from the ear, filtered in the second stage.

#### 2.2.2. Calculation of HRV and PTT Parameters

**Figure 10:**
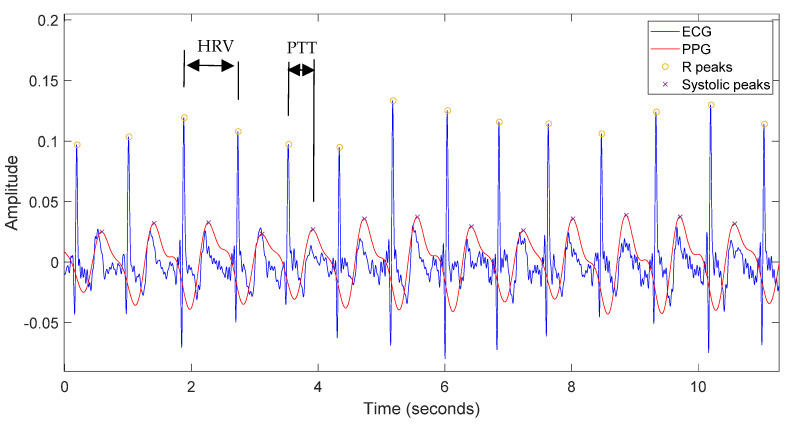
Filtered ECG signal (in blue) and filtered PPG signal (in red) for 10 s. HRV parameter is obtained from the ECG signal by calculating the time between the R-peaks. To calculate the PTT parameter, it is necessary to measure the time between the R-peak of the ECG signal and the next peak in the PPG signal.

Once the signals had been filtered, the heart rate variability (HRV) and the pulse transit time (PTT) were obtained from the ECG and PTT signals. As shown in Figure 10, the HRV parameter is obtained from the ECG signal by calculating the time between the R-peaks. In order to calculate the PTT parameter, it is necessary to measure the time between the R-peak of the ECG signal and the next peak in the PPG signal. In order to determine when these peak values are reached, the derivatives of both signals are calculated, and when they reach a certain pre-set threshold, a peak is detected. This can be performed because both the R-peak of the ECG signal and the maximum value of the PPG signal is reached with a steep slope due to the systolic phase of the cardiac cycle [71]. Figure 10 shows the filtered ECG signal (in blue) and the filtered PPG signal (in red) and the definition of the HRV and PTT parameters. Both of them are usually calculated in milliseconds.

### 2.3. Ambulatory System Validation

Once the static validation of the monitoring system had been carried out and the correct acquisition of the signals under study had been verified, the ambulatory validation of the system was performed. The aim of this validation was to assess the correct elimination of possible artifacts (movements) that may interfere with the proper acquisition of the involved signals (ECG, PPG, and ear EEG). For this purpose, recordings were made with 10 healthy volunteers (Table 2). The total duration of the ambulatory experimentation was set to 30 min, divided into the following everyday tasks:

1. **Have a snack *(3 min)*:** The user has a snack while sitting on the chair. During this activity, due to chewing, artifacts are likely to occur in the ear EEG signal.
2. **Brushing teeth *(3 min)*:** The user is standing while brushing his teeth. Due to hand movements, artifacts in the ECG and PPG signal may appear.
3. **Combing the hair *(3 min)***: The user is standing and combing their hair. Again, the hand movements can lead to artifacts in the ECG and PPG signal.
4. **Outdoor walk with concentration activity *(7 min)*:** The user takes a walk outdoors during the day. At the same time, the user has to memorize sequences of numbers told by another person while walking. The walking movement might yield artifacts in all signals. In addition, it could increase the user’s heart rate.
5. **Read a book in silence *(3 min)*:** The user is sitting and reading a book in a silent atmosphere. This activity is intended to keep the user relaxed and so that the signals are recorded free of artifacts.
6. **Power walking *(8 min)*:** The user goes for a walk again, but this time faster. This circumstance may increase both the signal artifacts due to movement and the heart rate.
7. **Doing a sudoku *(3 min)*:** The user is sitting and doing a sudoku. The aim of this activity is to keep the user concentrated and to so that a lower heart rate is registered. In addition, when performing a concentration task related to a mathematical problem, it should be possible to observe how beta waves predominate in the ear EEG signal.

#### 2.3.1. Signal Processing

Since the signals are acquired in an ambulatory environment where there are a number of events and circumstances that can potentially increase the noise-to-signal ratio, more sophisticated signal processing methods are required. The processing carried out to improve the quality of each of the signals studied (ECG, PPG, and ear EEG) is described below.

In order to improve the quality of the raw ECG signal recorded in ambulatory settings, a bandpass filter was implemented from 0.4 to 80 Hz. Then, an IIR (Infinite Impulse Response) notch filter was implemented to remove the noise associated with the alternating current (50 Hz). Later, Matlab’s Wavelet Toolbox [72] was used to perform a 10-level wavelet decomposition of the signal using the Daubechies wavelet of order 6 and then subtracting it from the original signal. Finally, a smoothing of the signal was performed. As for the PPG signal, a 1 to 2 Hz bandpass filter followed by a moving median filter of order 15 was implemented. Hence, small peaks caused by motion artifacts are eliminated, and it is possible to accurately calculate the systolic peaks in the PPG signal. In order to remove artifacts in the EEG signals from the ear, a bandpass filter was first implemented from 0.5 to 40 Hz, and the trend of the signals was subtracted to remove possible deviations. Then, the wavelet-enhanced ICA (wICA) method presented in [73] was applied. This algorithm is based on independent component analysis (ICA) and makes use of wavelet thresholding. Specifically, the algorithm follows the next steps:

1. Apply a conventional ICA decomposition to raw EEG, thus obtaining the mixing matrix M and N independent components {s_1_(t), s_2_(t),…,s_N_(t)}.
2. Wavelet transform components obtaining their representations {W(j, k)}s_i_.
3. Threshold the wavelet coefficients, i.e., set W(j, k) = 0 for those that are higher than the threshold, |W(j, k)| > K.
4. Inverse wavelet transform of the thresholded coefficients W(j, k) thus recomposing components consisting sources of the neural origin only {n_i_(t)}.
5. Compose wICA-corrected EEG: X (t) = M · [n_1_(t), n_2_(t), … , n_N_(t)]^T^.

After obtaining the processed EEG signals, they were segmented into 20-s time windows with an overlap of 50% in order to extract some parameters for each window from each signal. For each window, the following parameters were calculated: statistical features (mean, standard deviation, variance, skewness, and kurtosis), entropy, and the power spectrum density (PSD) (obtained by pburg [74] in Matlab). Furthermore, the three ear EEG channels were again bandpass filtered in a second stage to obtain information about the different waves that make up the signal (Delta, Theta, Alpha, and Beta waves). 

#### 2.3.2. Calculation of HRV and PTT Parameters

Due to artifacts originating in the ambulatory environment, it is possible that sometimes the noise may be so noticeable that it interferes with the integrity of the studied signals. In this case, false peaks are likely to be detected, such as spurious R-peaks in the ECG signal or systolic peaks in the PPG signal [75]. Therefore, it is necessary to implement a robust algorithm that allows us to discern when a false peak has been detected. In [76], an algorithm based on a time-varying threshold and a novel classification scheme capable of detecting abnormal heartbeats with high accuracy were presented. However, to the best of our knowledge, there are no existing algorithms that are simultaneously capable of accurately detecting and correcting erroneous HRV and PTT parameter values. We hereby propose one.

The proposed algorithm for calculating the HRV parameter consists of computing the maximums of the ECG signal by calculating the derivative of this signal (as shown in the static validation). The new value of each parameter is then calculated and checked to ensure its proper value. For this purpose, the algorithm checks that the new value shows a variation of a maximum of ±25% with respect to the previous value for the same parameter. If this condition is not met, the value of the previous HRV parameter is maintained.

Regarding the calculation of the PTT parameter, the algorithm consists of obtaining the maximums of the ECG and PPG signals by calculating the derivative of these signals in order to calculate a new PTT value. When detecting the peaks of both signals, it is highly probable that they do not coincide due to the existence of false or undetected peaks. If the value of this new parameter is greater than 700 milliseconds or smaller than 200 milliseconds (values outside the normal range in healthy patients), it means that a peak has been missed or a false peak has been detected. In this case, a local ECG/PPG peak readjustment is performed, and the PTT parameter is recalculated. Then, the algorithm verifies that the new parameter has a maximum variation of ±25% with respect to its previous value, as before. Again, if this condition is not met, the value of the previous PTT parameter is maintained. It should be noted that these maximum percentage variations can be customized and adjusted after calibration. 

**Figure 11:**
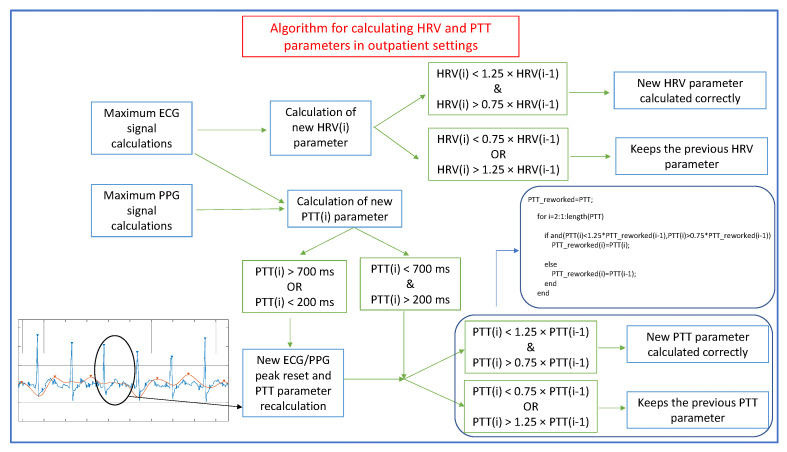
Schematic of the algorithm for the calculation of the HRV and PTT parameters in the ambulatory validation of the monitoring system. The lower left corner shows an example where a systolic peak is not detected in the PPG signal and therefore a local readjustment of the ECG (blue) and PPG (red) peaks is necessary.

Figure 11 depicts the algorithm for calculating the HRV and PTT parameters in outpatient settings. An inset showing the ECG and PPG signals is also shown, and an example can be observed where a systolic peak is not detected in the PPG signal, and, therefore, a local readjustment of the ECG and PPG peaks is necessary. In addition, the code devoted to checking that the new PTT parameter has a maximum variation of ±25% with respect to its previous value is also shown.

## 3. Results

The validation and comparison of the proposed device with the commercial Zephyr BioHarness and the e-Health platform under static conditions is shown below. In addition, the results of applying the artifact removal techniques to the recorded signals in the ambulatory setting are also shown. Furthermore, the variation of the parameters in the static and dynamic validation of the device will be observed.

### 3.1. Validation and Comparison of the Static System

**Figure 12:**
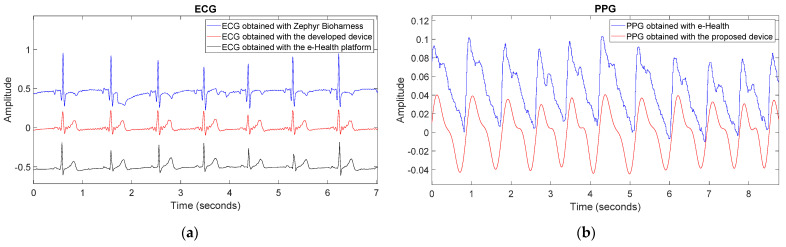
(**a**) ECG signal obtained with Zephyr BioHarness in blue, with-Health in dark and ECG with the proposed device in red; (**b**) PPG signal obtained with the e-Health pulse oximeter in blue and with the proposed device in red.

A comparison between the signals obtained with the proposed device and those from Zephyr BioHarness and e-Health board is shown in Figure 12. On the one hand, Figure 12a shows in blue the ECG signal obtained with Zephyr BioHarness (dry electrodes), in black the ECG signal obtained with e-Health (wet electrodes), and in red the signal obtained with the developed device. As a result, it can be seen that the quality of the obtained signals is very similar. The cross-correlation coefficient of the signal obtained with Zephyr BioHarness and the signal obtained with the proposed device is 0.70. Although there is no perfect correlation between the two signals, it can be observed that the systolic peaks coincide and are easily detectable. On the other hand, the cross-correlation coefficient of the ECG signal obtained with e-Health and the signal obtained with our device is 0.92, showing a significative correlation.

On the other hand, Figure 12b shows in blue the PPG signal obtained with the e-Health pulse oximeter and in red the signal obtained with the proposed device. This comparison results in a high similarity of both signals. The cross-correlation coefficient of the two signals is ∼0.96, meaning a high correlation. It is interesting to note that the signal from the proposed device is also suitable for recording the systolic peaks and even the dicrotic notches [77].

**Figure 13:**
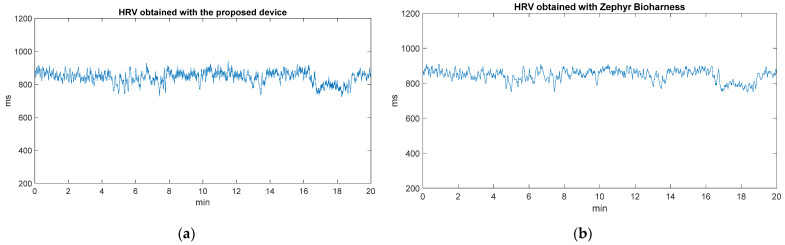
(**a**) Variation of HRV parameter values obtained with the proposed device; (**b**) Variation of HRV parameter values obtained with Zephyr Bioharness.

Concerning the assessment of the HRV parameter values obtained with the developed device, they are compared with the values provided by the Zephyr BioHarness in Figure 13. As a result, high accuracy is obtained by the proposed system. Figure 13a shows the variation of the HRV parameter values throughout the static experimentation, and Figure 13b shows the same variation of the HRV values recorded with Zephyr BioHarness. The mean absolute percentage error (MAPE) value is roughly 7%. According to [78], an MAPE of <10% was deemed reliable.

**Figure 14:**
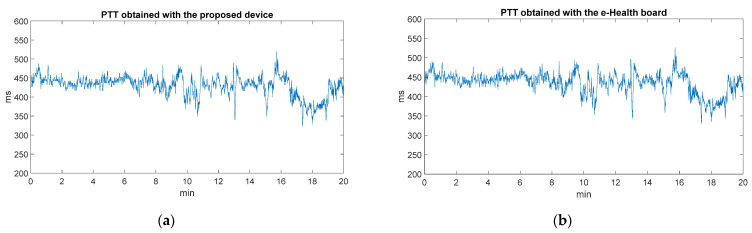
(**a**) Variation of PTT parameter values obtained with the developed device; (**b**) Variation of PTT parameter values obtained with Zephyr Bioharness.

The accuracy of the PTT values is determined by the quality of the acquired ECG and PPG signals (which have been previously validated and compared with other commercial systems) and their correct synchronization. Regarding the latter, correct synchronization was tested by acquiring ECG and PPG using the e-Health board and then calculating the PTT values. As a result, it can be observed that the variation of these values is very similar. Figure 14a shows the variation of the PTT values throughout the static experimentation with the proposed device, and Figure 14b shows the values recorded with the e-Health board. The mean absolute percentage error (MAPE) value is roughly 8%.

### 3.2. Removal of Artifacts in the ECG Signal in Outpatient Settings

**Figure 15:**
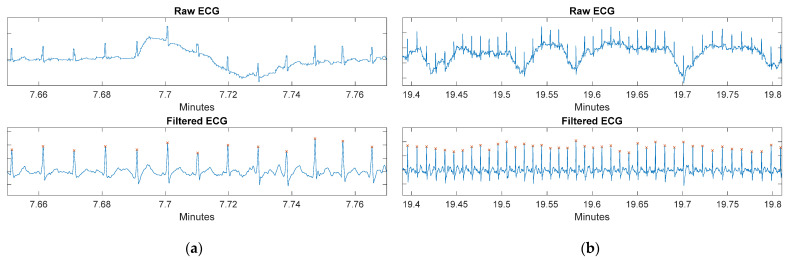
(**a**) Removal of a motion artifact while the subject was combing their hair. (**b**) Elimination of motion artifacts while the subject is walking fast.

The top of Figure 15a shows the raw acquired ECG signal, which is contaminated by a motion artifact caused while the subject was combing their hair (task 3). At the bottom, the correct removal of the artifact can be seen. Furthermore, at the top of Figure 15b, the ECG signal contaminated by several motion artifacts caused by body movements during task six is plotted. The bottom shows the correct elimination of these artifacts and the proper detection of the R-peaks. These plots show how the artifacts are removed by the filtering techniques described in the previous section, and how it is possible to obtain the exact location of the R-peaks in the ECG signal.

### 3.3. Removal of Artifacts in the PPG Signal in Outpatient Settings

**Figure 16:**
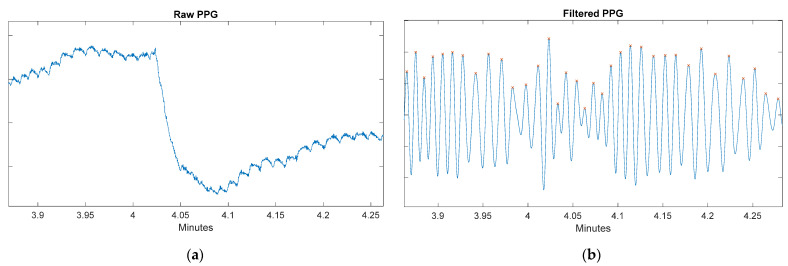
(**a**) Raw PPG signal during toothbrushing at the fourth minute; (**b**) Filtered PPG signal during toothbrushing at the fourth minute. The correct detection of the marked peaks can also be observed.

Figure 16a shows a motion artifact in the raw PPG signal due to the movement of the wrist while the user was brushing their teeth during the fourth minute (task 2), whilst Figure 16b shows the result of applying the signal processing techniques to the raw signal. It can be seen that although the signal loses the dicrotic notches, the location of the systolic peaks can be obtained, and, therefore, the heart rate and PTT parameters can be calculated.

### 3.4. Removal of Artifacts in the Ear EEG Signal in Outpatient Settings

**Figure 17:**
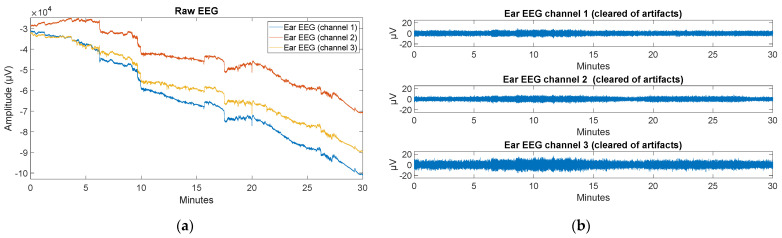
(**a**) Raw ear EEG signals; (**b**) Artifact-free ear EEG signals.

Figure 17a shows the raw recording of the three ear EEG channels. Here it can be seen that there is measurement drift as well as different artifacts throughout the 30 min of experimentation in the ambulatory environment. Figure 17b shows the result (free of artifacts) of applying the different processing techniques (including the wICA method) to the signals shown in Figure 17a.

**Figure 18:**
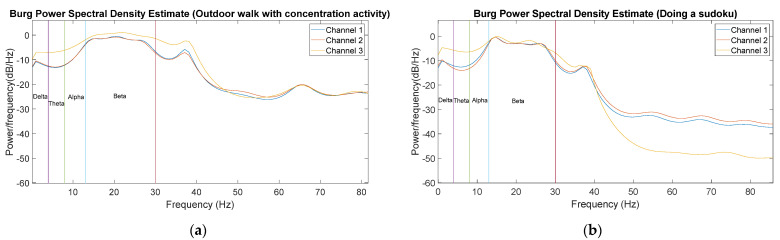
(**a**) Burg Power Spectral Density Estimate of the three ear EEG channels of a time window at around tenth minute (outdoor walk with concentration activity); (**b**) Burg Power Spectral Density Estimate of the three ear EEG channels of a time window at around 28th minute (doing sudoku).

Further processing these signals, Figure 18a shows the Burg Power Spectral Density Estimate of the three ear EEG channels of a 20 s window at around the tenth minute. This plot shows a power increase in the beta band because, at this point, the user was already performing the outdoor walk with concentration activity (task 4). Moreover, in Figure 18b, the Burg Power Spectral Density Estimate of the three ear EEG channels of a 20 s window at around the 28th minute can be observed. Since, at this moment of the experiment, the user was doing a sudoku (concentration activity, task 7), a power increase can be again observed in the beta band. A sharp power drop is also observed in both figures from 40 Hz onwards due to the bandpass filter up to this frequency.

### 3.5. Variation of HRV and PTT Parameters

**Figure 19:**
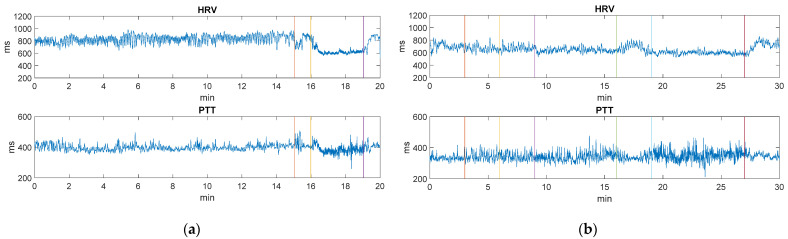
(**a**) Variation in milliseconds of HRV and PTT parameters in static validation. (**b**) Variation in milliseconds of HRV and PTT parameters in ambulatory validation. The vertical colored lines separate the periods of the different activities developed during the experimentation.

Figure 19a shows the variation in milliseconds of the HRV and PTT parameters in the static validation over the 20 min of the experiment. It can be seen how hyperventilation (16–19 min) produces a decrease in the values of the HRV and PTT parameters. This phenomenon was observed in all five cases under study due to the fact that hyperventilation causes an increase in power and heart rate [79].

Regarding the ambulatory validation, Figure 19b shows the variation in milliseconds of the HRV and PTT parameters that are calculated using the robust algorithm expected to discriminate the missed or false peaks. It can be seen how, from minutes 9 to 16, the value of the HRV parameter decreases (heart rate increases) because the user starts to walk. Then, when reading in silence (from minutes 16 to 19) the HRV value increases and when the user starts walking again, this time more quickly (19–27 min), these values decrease again. Finally, when the sudoku activity is carried out, and the user is seated again, the HRV values begin to increase. With regard to the values of the PTT parameter, as expected, there is no great variation during the whole experiment.

## 4. Discussion

In this section, the quality of the recorded signals will be discussed, and the results obtained statically will be compared with those obtained in an ambulatory environment. In addition, the variability of the HRV and PTT parameters calculated in an ambulatory environment will be compared with those calculated statically.

The statically recorded ECG signal has been obtained free of artifacts with an approximate SNR of 5 dB. With only a bandpass filter and subsequent smoothing, the signal seems to reach an acceptable quality (Figure 8a and Figure 10), reducing the SNR to values around 2 dB. Notwithstanding that, as the ECG signal is acquired in ambulatory mode and is contaminated by a multitude of artifacts (with an approximate SNR of 15 dB), further signal processing (bandpass filter, IIR notch filter, wavelet decomposition, and smoothing) is necessary. By performing such processing, it is possible to acquire a signal that is very close to the statically recorded signal in terms of quality (Figure 15).

As for the PPG signal, when obtained statically (free of artifacts) with an approximate SNR of 4 dB, it can be observed that with a bandpass filter, the signal remarkably improves (Figure 8b and Figure 10), reducing the SNR to values around 2 dB, obtaining enough quality to even discern the dicrotic notches. However, due to the movements in the wrist and fingers during the ambulatory validation, the signal quality worsens considerably, showing an SNR of approximately 17 dB. Therefore, a filtering stage (with lower bandwidth) followed by a moving median filter has been required. After this processing, it can be observed that, albeit the resulting signal loses information since the dicrotic notches can no longer be detected (Figure 16), the systolic peaks can be accurately discerned, and consequently, the heart rate or PTT parameter can be properly calculated. 

In the static validation, for each one of the 3 ear EEG signals obtained (with an approximate SNR of 10 dB), a notch filter first and a bandpass filter later were used. This procedure allows for eliminating drift and noise from the signal, resulting in a fairly clear signal (Figure 9). However, during the ambulatory validation, it was necessary to use other techniques (bandpass filter with lower bandwidth and the wICA method) in order to successfully remove all the new artifacts (Figure 17). Although the removal artifacts in the ear EEG signal (with an approximate SNR of 30 dB) also yielded some loss of information, the quality of the resulting signal was notably enhanced. In order to validate the acquired ear EEG signal, a power increase in the beta band was verified for concentration-demanding tasks (Figure 18a,b). As a result, it has been verified that the same results of the static case can be achieved during activities in an outdoor environment where there are spurious artifacts in the recorded signals.

When observing the variation of the HRV and PTT parameters in the static validation (Figure 19a), it can be seen how it is possible to accurately monitor the changes produced in these parameters. As for the results obtained in the ambulatory validation (Figure 19b), the results obtained are coherent with those recorded statically in terms of variability, thanks to the applied algorithm. This algorithm implies a certain loss of data in the calculation of the HRV and PTT parameters since if these values fall out of a determined range, they are eliminated. However, considering an average of 75 beats per minute, meaning 2250 HRV and PTT intervals, only around 25 intervals are lost, which represents a loss of approximately 1% of the total information during the experimentation. Therefore, it can be concluded that the algorithm does not lead to an excessive loss of information and it significantly improves the variability of the data in the ambulatory validation, reducing the variability of the values with respect to their adjacent mean values from 50% to less than 15%.

## 5. Conclusions

A novel monitoring system has been successfully developed for static and/or ambulatory use, which provides information about HRV and PTT parameters as well as statistical features, entropy, and the power spectrum density (PSD) of the ear EEG signals. Considering the biomedical variables that the proposed device is able to record, it can find applications for monitoring patients who suffer from epilepsy. It is worth highlighting the quality of the obtained signals (ECG, PPG, and ear EEG) by applying the different signal processing methods that have been described throughout the article, as well as the reliability and accuracy of the HRV and PTT parameter values achieved thanks to the robust algorithm that discriminates the miscalculated cases. 

Despite the fact that the developed device has been tested only with healthy patients, it can serve as a clinical decision support tool, as it has been validated and compared with other commercial and independent systems in a static way, obtaining very similar results. In addition, it has been proved the correct obtention of parameters (HRV, PTT and extracted characteristics from the ear EEG signal) that provide information about the users’ health status in ambulatory environments. Further advances in this technology can be expected by testing the device with unhealthy patients so that the detection capabilities can be precisely adjusted to the requirements of each specific case. The provided information could also serve as a basis for early detecting the onset of certain diseases based on the information provided by the device and the use of artificial intelligence techniques.

## Figures and Tables

**Table 1:** Information about the subjects in the static system validation.

Gender	Age (Years)	Weight (kg)
Male	26	78
Male	29	65
Female	24	63
Male	47	80
Female	23	65

**Table 2:** Information about the subjects in the ambulatory system validation.

Gender	Age (Years)	Weight (kg)
Male	24	90
Male	26	78
Female	25	65
Male	41	72
Female	23	65
Male	24	78
Male	47	80
Female	23	56
Female	24	63
Male	29	65

## Data Availability

Not applicable.

## References

[1] Baulac M., De Boer H., Elger C., Glynn M., Kälviäinen R., Little A., Mifsud J., Perucca E., Pitkänen A., Ryvlin P. (2015). Epilepsy priorities in Europe: A report of the ILAE-IBE, Epilepsy Advocacy Europe Task Force. Epilepsia.

[2] Sana F., Isselbacher E.M., Singh J.P., Heist E.K., Pathik B., Armoundas A.A. (2020). Wearable Devices for Ambulatory Cardiac Monitoring: JACC State-of-the-Art Review. J. Am. Coll. Cardiol..

[3] Kuehn B.M. (2016). Telemedicine helps cardiologists extend their reach. Circulation.

[4] Li H., Boulanger P. (2020). A Survey of Heart Anomaly Detection Using Ambulatory Electrocardiogram (ECG). Sensors.

[5] Rubio P., Hampel K., Giner P. (2020). Grafoelementos, artifactos e informe del EEG. Guía práctica de Epilepsia de la Comunidad Valenciana.

[6] Harrigan R.A., Chan T.C., Brady W.J. (2012). Electrocardiographic Electrode Misplacement, Misconnection, and Artifact. J. Emerg. Med..

[7] Serhani M.A., El Kassabi H.T., Ismail H., Nujum Navaz A. (2020). ECG Monitoring Systems: Review, Architecture, Processes, and Key Challenges. Sensors.

[8] Kaur J., Kaur A. A review on analysis of EEG signals. Proceedings of the International Conference on Advances in Computer Engineering and Applications.

[9] Karpiel I., Kurasz Z., Kurasz R., Duch K. (2021). The Influence of Filters on EEG-ERP Testing: Analysis of Motor Cortex in Healthy Subjects. Sensors.

[10] McDermott E.J., Raggam P., Kirsch S., Belardinelli P., Ziemann U., Zrenner C. (2022). Artifacts in EEG-Based BCI Therapies: Friend or Foe?. Sensors.

[11] Becker D.E. (2006). Fundamentals of Electrocardiography Interpretation. Anesthesia Prog..

[12] Park J., Seo H., Kim S.-S., Shin H. (2022). Photoplethysmogram Analysis and Applications: An Integrative Review. Front. Physiol..

[13] Castaneda D., Esparza A., Ghamari M., Soltanpur C., Nazeran H. (2018). A review on wearable photoplethysmography sensors and their potential future applications in health care. Int. J. Biosens. Bioelectron.

[14] Šumak B., Brdnik S., Pušnik M. (2020). Sensors and Artificial Intelligence Methods and Algorithms for Human–Computer Intelligent Interaction: A Systematic Mapping Study. Sensors.

[15] Suhaimi N.S., Mountstephens J., Teo J. (2020). EEG-Based Emotion Recognition: A State-of-the-Art Review of Current Trends and Opportunities. Comput. Intell. Neurosci..

[16] Brambilla C., Pirovano I., Mira R.M., Rizzo G., Scano A., Mastropietro A. (2021). Combined Use of EMG and EEG Techniques for Neuromotor Assessment in Rehabilitative Applications: A Systematic Review. Sensors.

[17] Cincotti F., Pichiorri F., Aricò P., Aloise F., Leotta F., de Vico Fallani F., Millán J.D.R., Molinari M., Mattia D. EEG-based Brain-Computer Interface to support post-stroke motor rehabilitation of the upper limb. Proceedings of the Annual International Conference of the IEEE Engineering in Medicine and Biology Society.

[18] Nafea M., Hisham A.B., Abdul-Kadir N.A., Harun F.K.C. Brainwave-Controlled System for Smart Home Applications. Proceedings of the 2nd International Conference on BioSignal Analysis, Processing and Systems (ICBAPS).

[19] Kumari P., Vaish A. (2015). Brainwave based user identification system: A pilot study in robotics environment. Robot. Auton. Syst..

[20] Katona J., Ujbanyi T., Sziladi G., Kovari A. Speed control of Festo Robotino mobile robot using NeuroSky MindWave EEG headset based brain-computer interface. Proceedings of the 7th IEEE International Conference on Cognitive Infocommunications (CogInfoCom).

[21] Katona J., Kovari A. (2018). The Evaluation of BCI and PEBL-based Attention Tests. Acta Polytech. Hung..

[22] Katona J., Ujbanyi T., Sziladi G., Kovari A. Examine the effect of different web-based media on human brain waves. Proceedings of the 8th IEEE International Conference on Cognitive Infocommunications (CogInfoCom).

[23] Kasprowski P., Harezlak K., Niezabitowski M. Eye movement tracking as a new promising modality for human computer interaction. Proceedings of the 17th International Carpathian Control Conference (ICCC).

[24] Katona J. (2021). Analyse the Readability of LINQ Code using an Eye-Tracking-based Evaluation. Acta Polytech. Hung..

[25] Katona J. (2022). Measuring Cognition Load Using Eye-Tracking Parameters Based on Algorithm Description Tools. Sensors.

[26] Smith S.J.M. (2005). EEG in the diagnosis, classification, and management of patients with epilepsy. J. Neurol. Neurosurg. Psychiatry.

[27] Zhou M., Tian C., Cao R., Wang B., Niu Y., Hu T., Guo H., Xiang Z. (2018). Epileptic Seizure Detection Based on EEG Signals and CNN. Front. Neuroinform..

[28] Meiser A., Tadel F., Debener S., Bleichner M.G. (2020). The Sensitivity of Ear-EEG: Evaluating the Source-Sensor Relationship Using Forward Modeling. Brain Topogr..

[29] Athavipach C., Pan-Ngum S., Israsena P. (2019). A Wearable In-Ear EEG Device for Emotion Monitoring. Sensors.

[30] Bleichner M.G., Debener S. (2017). Concealed, Unobtrusive Ear-Centered EEG Acquisition: cEEGrids for Transparent EEG. Front. Hum. Neurosci..

[31] Zibrandtsen I.C., Kidmose P., Christensen C.B., Kjaer T.W. (2017). Ear-EEG detects ictal and interictal abnormalities in focal and gener-alized epilepsy—A comparison with scalp EEG monitoring. Clin. Neurophysiol..

[32] Gordan R., Gwathmey J.K., Xie L.-H. (2015). Autonomic and endocrine control of cardiovascular function. World J. Cardiol..

[33] DeGiorgio C.M., Miller P., Meymandi S., Chin A., Epps J., Gordin S. (2010). RMSSD, a measure of vagus-mediated heart rate variability, is associated with risk factors for SUDEP: The SUDEP-7 Inventory. Epilepsy Behav..

[34] Singh N., Moneghetti K.J., Christle J.W., Hadley D., Plews D., Froelicher V. (2018). Heart Rate Variability: An Old Metric with New Meaning in the Era of using mHealth Technologies for Health and Exercise Training Guidance. Part One: Physiology and Methods. Arrhythm. Electrophysiol. Rev..

[35] Moridani M.K., Farhadi H. (2017). Heart rate variability as a biomarker for epilepsy seizure prediction. Bratisl. Med. J..

[36] Myers K.A., Bello-Espinosa L.E., Symonds J.D., Zuberi S.M., Clegg R., Sadleir L.G., Buchhalter J., Scheffer I.E. (2018). Heart rate variability in epilepsy: A potential biomarker of sudden unexpected death in epilepsy risk. Epilepsia.

[37] Block R.C., Yavarimanesh M., Natarajan K., Carek A., Mousavi A., Chandrasekhar A., Kim C.-S., Zhu J., Schifitto G., Mestha L.K. (2020). Conventional pulse transit times as markers of blood pressure changes in humans. Sci. Rep..

[38] Smith R.P., Argod J., Pépin J.-L., Lévy P.A. (1999). Pulse transit time: An appraisal of potential clinical applications. Thorax.

[39] Nass R.D., Hampel K., Elger C.E., Surges R. (2019). Blood Pressure in Seizures and Epilepsy. Front. Neurol..

[40] Post-ictal Physiology: Adding Blood Pressure to the Equation. https://www.epilepsy.com/article/2016/12/post-ictal-physiology-adding-blood-pressure-equation.

[41] Singh A., Hussain A.A., Lal S., Guesgen H.W. (2021). A Comprehensive Review on Critical Issues and Possible Solutions of Motor Imagery Based Electroencephalography Brain-Computer Interface. Sensors.

[42] Diykh M., Li Y., Wen P., Li T. (2018). Complex networks approach for depth of anesthesia assessment. Measurement.

[43] Covantes-Osuna C., López J.B., Paredes O., Vélez-Pérez H., Romo-Vázquez R. (2021). Multilayer Network Approach in EEG Motor Imagery with an Adaptive Threshold. Sensors.

[44] Apple, Why Apple Watch. https://www.apple.com/watch/why-apple-watch/.

[45] iRHYTHM Technologies, Uninterrumpled Ambulatory Cardiac Monitoring. https://www.irhythmtech.com/.

[46] Integrated M. MAX-ECGMONITOR Wearable ECG and Heart Monitor Evaluation and Development Platform. https://www.maximintegrated.com/en/products/interface/sensor-interface/MAX-ECGMONITOR.html.

[47] Medtronic Zephyr Performance Systems. https://www.zephyranywhere.com.

[48] Fitbit, Advanced Fitness + Health Tracker. https://www.fitbit.com/global/us/products/trackers/charge5.

[49] Cosinuss, «cosinuss One—Performance Monitoring. https://www.cosinuss.com/en/products/data-acquisition/in-ear-sensors/one/.

[50] Medtronic, Nellcor™ Portable SpO₂ Patient Monitoring System. https://www.medtronic.com/covidien/en-us/products/pulse-oximetry/nellcor-portable-spo2-patient-monitoring-system.html.

[51] Oura Health, Accurate Health Information Accesible to Everyone. https://ouraring.com/.

[52] Emotiv Epoc Flex—32-Channel Wireless EEG Device. https://www.emotiv.com/epoc-flex/.

[53] NeuroSky MindWave. https://store.neurosky.com/pages/mindwave.

[54] Tmsi EEG Headcaps. https://www.tmsi.com/products/eeg-headcaps/.

[55] MJN Seras. https://mjn.cat/.

[56] Masihi S., Panahi M., Maddipatla D., Hanson A.J., Fenech S., Bonek L., Sapoznik N., Fleming P.D., Bazuin B.J., Atashbar M.Z. (2021). Development of a Flexible Wireless ECG Monitoring Device with Dry Fabric Electrodes for Wearable Applications. IEEE Sensors J..

[57] Kim B.H., Jo S., Choi S. (2021). ALIS: Learning Affective Causality Behind Daily Activities from a Wearable Life-Log System. IEEE Trans. Cybern..

[58] Juez J., Henao D., Segura F., Gomez R., Le Van Quyen M., Valderrama M. Development of a wearable system with In-Ear EEG electrodes for the monitoring of brain activities: An application to epilepsy. Proceedings of the IEEE 2nd International Congress of Biomedical Engineering and Bioengineering (CI-IB&BI).

[59] Yamakawa T., Miyajima M., Fujiwara K., Kano M., Suzuki Y., Watanabe Y., Watanabe S., Hoshida T., Inaji M., Maehara T. (2020). Wearable Epileptic Seizure Prediction System with Machine-Learning-Based Anomaly Detection of Heart Rate Variability. Sensors.

[60] OpenBCI. www.openbci.com.

[61] Ahufinger S., Balugo P., González M.M., Pequeño E., González H., Herrero P. (2019). A User-centered Smartphone Application for Wireless EEG and its Role in Epilepsy. IJIMAI.

[62] Peterson V., Galván C., Hernández H., Spies R. (2020). A feasibility study of a complete low-cost consumer-grade brain-computer interface system. Heliyon.

[63] Rashid U., Niazi I.K., Signal N., Taylor D. (2018). An EEG Experimental Study Evaluating the Performance of Texas Instruments ADS1299. Sensors.

[64] MAX86150 Datasheet. https://datasheets.maximintegrated.com/en/ds/MAX86150.pdf.

[65] Golden D.P., Wolthuis R.A., Hoffler G.W. (1973). A Spectral Analysis of the Normal Resting Electrocardiogram. IEEE Trans. Biomed. Eng..

[66] Johnstone J.A., Ford P.A., Hughes G., Watson T., Garrett A.T. (2012). Bioharness(™) multivariable monitoring device: Part I: Validity. J. Sports Sci. Med..

[67] Johnstone J.A., Ford P.A., Hughes G., Watson T., Garrett A.T. (2012). Bioharness(™) Multivariable Monitoring Device: Part. II: Reliability. J. Sports Sci. Med..

[68] e-Health Sensor Platform V1.0 for Arduino and Raspberry Pi [Biometric/Medical Applications]. E-Health—Sensors—Shop. cooking-hacks.com.

[69] Biswas B.C., Bhalerao S.V. A real time based wireless wearable EEG device for epilepsy seizure control. Proceedings of the International Conference on Communications and Signal Processing (ICCSP).

[70] Lee M., Song C.B., Shin G.H., Lee S.W. (2019). Possible Effect of Binaural Beat Combined with Autonomous Sensory Meridian Response for Inducing Sleep. Front. Hum. Neurosci..

[71] Zambrana-Vinaroz D., Vicente-Samper J.M., Juan C.G., Esteve-Sala V., Sabater-Navarro J.M. (2019). Non-Invasive Device for Blood Pressure Wave Acquisition by Means of Mechanical Transducer. Sensors.

[72] Wavelet Toolbox (Matlab). https://es.mathworks.com/products/wavelet.html.

[73] Castellanos N., Makarov V.A. (2006). Recovering EEG brain signals: Artifact suppression with wavelet enhanced independent component analysis. J. Neurosci. Methods.

[74] Autoregressive Power Spectral Density Estimate—Burg’s Method. https://es.mathworks.com/help/signal/ref/pburg.html.

[75] Citi L., Brown E.N., Barbieri R. (2012). A real-time automated point-process method for the detection and correction of erroneous and ectopic heartbeats. IEEE Trans Biomed. Eng..

[76] Lipponen J.A., Tarvainen M.P. (2019). A robust algorithm for heart rate variability time series artefact correction using novel beat classification. J. Med. Eng. Technol..

[77] Hoeksel S.A., Jansen J.R., Blom J.A., Schreuder J.J. (1997). Detection of dicrotic notch in arterial pressure signals. J. Clin. Monit..

[78] Jachymek M., Jachymek M.T., Kiedrowicz R.M., Kaźmierczak J., Płońska-Gościniak E., Peregud-Pogorzelska M. (2022). Wristbands in Home-Based Rehabilitation—Validation of Heart Rate Measurement. Sensors.

[79] Wood C., Cano V.A. (2021). La Hiperventilación y el Trastorno de Angustia a la Luz de un Marco Cognitivo. Clín. Salud.

